# High-Precision Solvent Vapor Annealing for Block Copolymer Thin Films

**DOI:** 10.3390/mi9060271

**Published:** 2018-05-29

**Authors:** Gunnar Nelson, Chloe S. Drapes, Meagan A. Grant, Ryan Gnabasik, Jeffrey Wong, Andrew Baruth

**Affiliations:** Department of Physics, College of Arts and Sciences, Creighton University, 2500 California Plaza, Omaha, NE 68178, USA; GunnarNelson@Creighton.edu (G.N.); ChloeDrapes@Creighton.edu (C.S.D.); MeaganGrant@Creighton.edu (M.A.G.); gnaba001@umn.edu (R.G.); JeffreyWong@Creighton.edu (J.W.)

**Keywords:** block polymers, self-assembly, thin films, solvent vapor annealing, nanolithography

## Abstract

Despite its efficacy in producing well-ordered, periodic nanostructures, the intricate role multiple parameters play in solvent vapor annealing has not been fully established. In solvent vapor annealing a thin polymer film is exposed to a vapor of solvent(s) thus forming a swollen and mobile layer to direct the self-assembly process at the nanoscale. Recent developments in both theory and experiments have directly identified critical parameters that govern this process, but controlling them in any systematic way has proven non-trivial. These identified parameters include vapor pressure, solvent concentration in the film, and the solvent evaporation rate. To explore their role, a purpose-built solvent vapor annealing chamber was designed and constructed. The all-metal chamber is designed to be inert to solvent exposure. Computer-controlled, pneumatically actuated valves allow for precision timing in the introduction and withdrawal of solvent vapor from the film. The mass flow controller-regulated inlet, chamber pressure gauges, in situ spectral reflectance-based thickness monitoring, and low flow micrometer relief valve give real-time monitoring and control during the annealing and evaporation phases with unprecedented precision and accuracy. The reliable and repeatable alignment of polylactide cylinders formed from polystyrene-*b*-polylactide, where cylinders stand perpendicular to the substrate and span the thickness of the film, provides one illustrative example.

## 1. Introduction

Techniques to achieve periodic nanostructures via traditional “top down” methods, including photolithography, have become increasingly challenging within the semiconductor industry. Ultra-small feature production is approaching fundamental resolution limits (193 nm ultraviolet (UV) lithography, for example, recently reaching sub-30 nm features) [1,2,3,4,5,6,7,8,9,10,11]. One promising strategy is investigating “bottom up” approaches that rely on nanoscale self-assembly. In 2007, directed self-assembly was first considered as a potential scaling solution, according to the International Technology Roadmap for Semiconductors (ITRS) [11]. In their 2013 report, the directed self-assembly of complex structures with low anneal time, low defect density, and high reproducibility was identified as one of the “Grand Challenges” to extend Moore’s law [11]. The directed self-assembly of block polymer (BP) thin films has become a particularly strong candidate to achieve sub-20 nm dimensions, where the size and morphology is controlled by varying the molecular weight of the constituent polymer blocks.

Due to thorough investigations over decades, the BP research community generically considers the bulk behavior of many BPs well known [12,13]. Bulk morphologies are characterized by the Flory–Huggins interaction parameter (χ), the degree of polymerization (N), the volume fractions of the constituent blocks (f), and the particular architecture [13]. Furthermore, much is known about bulk behavior under a variety of stimuli, whether thermal, solvent, or mechanical [12]. In particular, measured or known quantities can well predict the alignment of structures and morphologies within the bulk. On the contrary, the confinement of a thin film and the associated surface energy contributions introduces additional confounders [14,15]. In particular, the complexity of surface interactions (both with the substrate and free surface) impose new, often asymmetric, boundary conditions [16,17]. Regardless, techniques designed to promote ordering of BP thin films have progressed rapidly, driven by applications requiring long-range lateral ordering, uniformity in feature size and high placement precision. Most of the historical approaches to BP ordering have been largely unpredictable and are often slow or energy-intensive: including thermal annealing [18,19], electric field alignment [20,21], and incorporation of low surface energy midblocks [22,23]. More recently, the use of pre-patterned substrates (chemical or topographical) to act as a guide for BP assembly has been successfully incorporated but can be time-intensive and involves multiple lithographic approaches [24]. Therefore, it is not necessarily cost-effective for high-throughput applications. As a result, a maturing technique is solvent vapor annealing (SVA) [25].

SVA was originally introduced as an alternative to thermal annealing for BPs exhibiting thermal degradation, problematic thermally-driven transitions, or slow dynamics due to high molar mass [26,27,28]. The interest in SVA of BPs has grown well beyond this in recent years. It has been shown to optimize organization quickly due to the increased chain mobility, a possibly decreased χ (dependent on solvent polarity), and tunable surface energies [25]. In this process, a BP film is exposed to the vapors of one or more organic solvents, offering direct control over lyotropic transitions (cylinders to spheres, for example) while in the solvated state as well as during evaporation [29,30]. This technique also has the ability to reduce defect density dramatically [31], while improving lateral ordering, both at the free surface and into the bulk of a film. This ordering is achieved more quickly (by several orders of magnitude) and completely than previous methods [32]. Although much is known about the interactions of BPs with solvent in bulk [33], the effects in thin films exhibit different behaviors due to the presence of confining surfaces and the dynamic exchange with the solvent vapor atmosphere [17]. Thus, this technique continues to suffer from reliability problems and no standardized methods have become apparent. It is becoming increasingly clear that a continued understanding of the specific ordering mechanisms of a BP system is paramount, where the quest for generic understanding of any BP thin film system remains elusive.

Of critical importance to many technological applications is the ability to direct BP thin films to form cylinders that stand perpendicular to the surface, traverse through the thickness of the film, and laterally pack with hexagonal order. A recently demonstrated approach to nanolithography of a magnetic thin film, for example, utilized such BP-based lithography masks using a Damascene-like approach. This approach was able to synthesize hexagonally-packed magnetic nanodots with a diameter of 19 nm with high fidelity and retention of robust ferromagnetism [34]. Furthermore, this approach achieved diameter control, down to 14 nm, and the potential for high-temperature processing with an additional atomic layer deposition step of ZnO [35]. These techniques rely on the vertical alignment of cylinders. On the contrary, many current advances in BP alignment, including important work in solvent concentration gradients [36], have focused solely on the free surface of the film. This is insufficient to be a direct substitution for most traditional lithographic approaches [37]. Recent results reveal that optimized ordering of hexagonally-packed cylinders can potentially occur in seconds and extend through the film using SVA, but this approach can be somewhat unreliable [38], where near 100% success of forming the desired morphology has yet to be achieved. Advances in computer simulation as well as in situ X-ray and neutron scattering continue to both further understand this process and improve upon it [39,40,41]. While such an understanding of the self-assembly behavior is critical to the advancement of this field, utilizing a statistical approach to quantifying SVA with large sample sets, with a goal of overcoming reliability barriers, requires the ability to identify, measure and develop controls for all of the pertinent variables with reliable precision. This includes chamber pressure, solvent exposure time, solvent concentration in the film, solvent evaporation rate, solvent purity or combinations, ambient temperature, sample and solvent temperature, humidity [42], and film thickness to name a few. These are addressed in the present manuscript, with a primary focus on the role of controlling chamber pressure and solvent evaporation rates at fast time scales of ~10 ms. We note that this chamber does not actively control sample [41] or solvent temperature [43,44,45], or include multiple solvents [46], which have been shown important to controlling annealing kinetics. So, these results work to keep these parameters as consistent as possible. To that end, we present here our strict annealing protocols and our climate-controlled SVA chamber with computer-controlled solvent vapor flow and pressure management and in situ spectral reflectance-based solvent concentration (φ) measurements. This chamber allows us to fix potential variables while investigating only one. This leads to unprecedented control over the SVA process with a goal of systematic studies with high reliability and repeatability that may have advantages as BP SVA alignment moves from its current research phase (relying heavily on in situ X-ray and neutron scattering) into scaled-up processing. One critical and unprecedented advancement is the ability to stabilize solvent concentration within a BP thin film for an arbitrarily long time at a user-defined chamber pressure, a necessary prerequisite for any temporal studies of crystallization.

**Figure 1:**
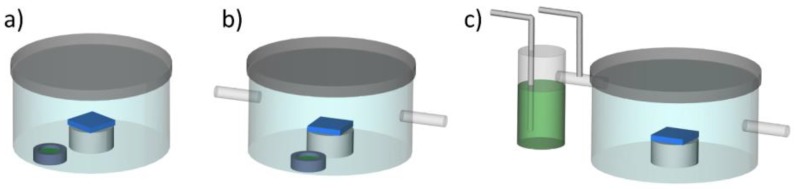
Primary methods of solvent vapor annealing; (**a**) “jar annealing,” where a sealed chamber contains the sample and a solvent reservoir; (**b**) “jar annealing” with inlet/outlet lines for inert gas flow; (**c**) solvent vapor flow via a carrier gas through inlet/outlet lines. Block polymer films are depicted in blue, with liquid solvent depicted in green.

To date, there are three primary methods of controlled SVA in the literature with differing levels of complexity, as illustrated in Figure 1. These processes were recently reviewed by Posselt et al. and Gu et al. [32,47], where each method typically incorporates an optical interferometer to monitor thickness (i.e., swelling due to solvent uptake) in real time that is placed above the chamber and shines down on the sample surface. Briefly, in “jar annealing,” depicted in Figure 1a, a solvent reservoir is placed in a sealed vessel with the BP film. The solvent vapor pressure, and thus the film swelling, is parameterized by the ratio of liquid solvent surface area to the chamber volume [48]. The solvent evaporation is either done via opening the lid, which is difficult to quantify, or introducing a leak until all solvent has evaporated [48,49]. A natural extension of this SVA method is the inclusion of inlet/outlet flow lines, as seen in Figure 1b, where the incorporation of an inert gas flow can serve to modify the vapor pressure and BP film swelling more directly [50]. However, there is still only limited control over the solvent evaporation rate. The next extension is displacing the solvent reservoir from inside the sealed chamber into a separate sealed reservoir, as shown in Figure 1c. In this SVA method, solvent is carried in the vapor phase by a carrier gas into the chamber. Both the absolute pressure and vapor pressure inside the chamber are controlled via the flowrates of the vapor line and a second inlet for an inert gas stream. This gives significant dynamic control of BP film swelling, although the highest achievable vapor pressure is reduced compared to Figure 1a,b due to a dilution of vapor from the carrier gas. The solvent evaporation rate is controlled in a similar fashion to the method depicted in Figure 1b, but without the presence of the solvent reservoir, thus offering more control. Similar methods have proliferated [30,51,52,53], but only few utilize real-time computer control [41,45] and there is currently an inability to maintain a fixed solvent concentration for an arbitrarily long time scale. The present manuscript describes a new evolution in the SVA method that builds on these existing models, adding additional control over solvent introduction into and evaporation out of a BP film with a high degree of reliability and reproducibility by controlling the vapor pressure through inlet/outlet flows.

Several recent in situ studies utilizing Grazing Incidence Small Angle X-ray and Neutron Scattering (GISAXS and GISANS) serve to motivate the ability to stabilize a BP film with a specific solvent concentration [41,54]. These studies have shown to be critical in identifying the role of the order-to-disorder transition in directed self-assembly, as well as the role of solvent polarity and temperature. Their results could be further extended with an increased level of control over solvent concentration during the anneal and during the solvent evaporation. For example, Gu et al. show a high degree of ordering in poly(styrene)-*b*-poly(2-vinyl pyridine) using GISAXS with an increasing solvent concentration [30]. Upon rapid evaporation, scanning electron micrographs (SEM) of the resultant films exhibit long correlation lengths of in-plane cylinders. Furthermore, recent results indicate that approaching a solvent concentration consistent with the order-to-disorder transition leads to the largest correlation lengths [32]. This solvent concentration is also consistent with enhanced defect removal [31]. Annealing at solvent concentrations above the order-disorder transition will, instead, result in a potentially disordered film, as previously shown [34]. In addition, Sinturel et al. revealed similar ordering in a solvated poly(styrene)-*b*-poly(lactide) film, where GISAXS was monitored with increasing solvent concentration [29]. The data shows that the correlation length of perpendicularly aligned cylinders in quickly dried films is largest for a high solvent concentration (below the order-to-disorder transition). Finally, Berezkin et al. recently showed how mixed solvents and the use of elevated temperature in a controlled SVA chamber (similar to Figure 1c) further enhance control over final morphologies. These GISAXS studies, while extremely insightful, rely on somewhat uncontrolled solvent exposure methods, as presented in Figure 1. In each case, no static solvent concentration (often described as swelling ratio) was achieved over any appreciable amount of time. Such control is a prerequisite for any temporal study of ordering kinetics and further optimization of an annealing protocol. Moving forward, especially when considering scaling up these techniques towards mass production, creating a highly controlled SVA chamber could assist in avoiding the use of continued in situ X-ray or neutron scattering techniques that are not easily extensible to scaling up. Instead, a highly controlled SVA chamber could establish reliability and repeatability that incorporates the critical parameters that have been established from these scattering techniques.

In addition to a controlled static solvent concentration, the evaporation rate serves as another complicating factor to be addressed. Solvent evaporation rate has been shown to strongly affect the surface morphology of BP films [52], where the evaporation has been shown to produce an ordering front that propagates through the film from the free surface to the substrate [55]. The timing of solvent removal dominates this effect. Enviable results were reported in 2004 from Kim et al. revealing defect-free ordering of cylinders at the free surface over large lateral length scales, where GISAXS data aimed to expose the role of this ordering front [55]. The ordering depends strongly on the exact trajectory through the BP phase diagram as solvent is removed [56], with changes in polymer dynamics and order–order transitions serving as potential complicating factors [57]. As such, conflicting results dictate that either slow [51,52] or fast [58] evaporation times may lead to ideal morphologies. Recently, studies of solvent concentration profiles [58] have suggested that the cylinder growth rate during evaporation is a product of the polymer chain mobility and the driving force to phase separate. Dynamical field theory simulations from Paradiso et al. further expose the role of rapid solvent evaporation and segregation strength (χ) on cylinder orientation [57]. It is increasingly clear that the ability to systematically remove solvent from the film, including at rapid time scales, is important to understanding final film morphologies. In particular, revealing how a given morphology propagates into the film during solvent evaporation, which is a necessary requirement for many nanolithographic applications. It is important to note that the role of solvent removal on rapid time scales (less than 100 ms) has not been successfully monitored using GISAXS, where integration times can be somewhat long (1–10 s) [32]; however, recent use of charge-coupled device (CCD) cameras have been shown to be effective for studying fast kinetics [47]. This opens the door for quantifying the effects of solvent removal in real time. Presently, in many of these studies, combining known structural properties from GISAXS in the solvated state with final morphologies imaged with atomic force microscopy (AFM) or SEM following rapid evaporation, as addressed above, have been used to reveal important relationships between evaporation trajectories and final morphologies. The present manuscript describes a systematically improved SVA method that offers a potential to elucidate multiple key variables in the SVA process and offers a level of control, reliability and reproducibility to enhance the understanding of ordering kinetics both during annealing and, critically, evaporation. Specifically, this improved SVA method monitors and controls chamber pressure, solvent exposure time, solvent concentration in the film, solvent purity and solvent evaporation rate, while concurrently monitoring ambient temperature and humidity.

## 2. Materials and Methods

### 2.1. Solvent Vapor Annealing

Optically polished substrates were single-crystalline silicon <111> wafers (n-type, 5 Ω·cm) with a native oxide layer (University Wafer, South Boston, MA, USA). No attempt was taken to remove the native oxide. Toluene (ACS Certified), tetrahydrofuran (THF) (Certified, contains 0.025% butylated hydroxytoluene as a preservative), acetone (99.5%), methanol (ACS Certified), and sodium hydroxide granules were all purchased from Fisher Chemical, Pittsburgh, PA, USA. 1,1,1,3,3,3-Hexamethyldisilazane (HMDS) (98%) and 3 Å, 4 to 8 mesh, 3333 were purchased from Acros Organics, Belgium, WI, USA.

Construction of the solvent vapor-annealing chamber (SAC) required chemically inert stainless-steel tubing and valves with Swagelok tube fittings. Pneumatically actuated stainless steel ball valves (1/4″ and 3/4″) utilizing Dow Corning M111 (heavy-consistency dimethyl silicone compound) lubricant and Modified PTFE packing (SS-T12-S-065-20 and SS-45S8-33C), stainless steel quarter-turn plug valves (1/4″) with Kalrez O-rings (SS-4P4T), a stainless steel low-flow metering valve (1/4”) utilizing a Kalrez O-ring (SS-SS4-KZ-VH), a stainless steel low-pressure (5 psig/34.5 kPa) unfilled pressure gauge (LP1-SS-254-5PSI), a stainless steel unfilled pressure gauge (60 psig/413.7 kPa, PGI-63C-PG60-LAOX), and 1/4” and 3/4” stainless steel tubing (SS-T4-S-035-20 and SS-T12-S-065-20, respectively) were purchased from the Swagelok Company, Saarland, OH, USA. The body and bubbler portions of the SAC utilized vacuum-grade Conflat flanges with oxygen-free copper gaskets. Stainless steel tees (275-150-CFT), 6-way cube (275-CUBE-OS), and all Conflat blanks (275-000-T) and Swagelok adaptors were purchased from LDS Vacuum Products, Inc., Longwood, FL, USA, or modified in-house. Conflat flanged zero length deep UV quartz (Corning HPFS 7980 Fused Silica) viewport was purchased from the Kurt J. Lesker Company, Jefferson Hills, PA, USA (VPZL-275Q). Dry N_2_ inlet was achieved with a Drierite gas purifier, and was controlled via an Apex 500 SCCM mass flow controller with RS-232 digital control (Schoonover Inc., Canton, GA, USA). Dry N_2_ pressure from the gas tank were monitored and controlled with a dual-stage gas regulator (0–344.7 kPa) and flowrates were monitored and controlled with a Panel-Mount Flowmeter (OMEGA Engineering, Stanford, CT, USA) for air, with a brass valve, with two different flow ranges (3–30 SCFH and 30–300 SCFH) from McMaster-Carr, Elmhurst, IL, USA. Pneumatic valve control was achieved with 3-way 1/4” NPT, normally closed, 120 V, 100 psi solenoids, various One-touch fittings (1/4”) and 1/4” nylon tubing purchased from the Swagelok Company, Saarland, OH, USA. Electric control of pneumatics was achieved with a National Instruments USB, 8 input, 12-Bit, 10 kS/s, Multifunction DAQ; 25 A, 250 V solid state relays; and LabVIEW 2016 software (National Instruments, Austin, TX, USA). Finally, film thickness was determined in situ with spectral reflectance via a Filmetrics F20-UV, San Diego, CA, USA, general-purpose film thickness measurement system with both halogen and deuterium sources.

### 2.2. Synthesis of Poly(Styrene)-Block-Poly(Lactide)

The synthesis of poly(styrene)-*block*-poly(lactide) (PS-*b*-PLA) is described fully elsewhere [34]. Succinctly, hydroxyl-terminated PS (Mn = 42.5 kDa) was synthesized via living anionic polymerization. The subsequent PLA was synthesized via ring opening transesterification polymerization (ROTEP) of d,l-lactide in dichloromethane at room temperature using 1,8-diazabicyclo[5.4.0]undec-7-ene (DBU) as a catalyst for 1 h. PS-*b*-PLA was obtained by precipitating in methanol after termination with benzoic acid. The final PS-*b*-PLA had a total Mn = 63 kDa, with a PLA volume fraction of 0.28 (by volume) yielding a cylindrical morphology with a polydispersity index of 1.08, as determined with ^1^H Nuclear Magnetic Resonance (NMR) and Size-Exclusion Chromatography (SEC) [34].

### 2.3. Thin Film Preparation

Typical solutions of 1.5% (w/v) PS-*b***-**PLA in toluene (non-selective solvent) were spin coated onto HMDS treated, natively-oxidized silicon wafers (20 mm × 20 mm). HMDS treatment of the Si wafers was carried out by ultrasonically cleaning substrates in organic solvents (acetone followed by methanol), treating them in a 1:5 (v/v) HMDS:toluene solution for 16 h, then rinsing in toluene to remove any excess HMDS that had not grafted fully to the substrate, and blowing dry with N_2_ gas. Treated wafers were then placed in a 75 °C oven to remove any residual solvent. The films were spin coated at 1000–3000 rpm for 30 s, diced into 12–16 pieces (~5 mm × 5 mm), and immediately placed in a 75 °C (below the glass transition temperature of either block [34]) oven for drying. This process yielded ~60 nm thick films, dependent on exact spin speed and solution concentration. Samples were dried for a minimum of one day. Periodic atomic force micrographs indicate no apparent aging issues or annealing effects while storing films at this temperature.

For the present experiment, thin films were hot-loaded into a N_2_ purged, over-pressurized solvent annealing chamber and immediately sealed to avoid water contamination. The samples were allowed to cool to room temperature and no further temperature control was implemented. This is described in full detail below. THF liquid was stored in a sealed Erlenmeyer flask over activated 3 Å molecular sieves for several days before introducing it to the SAC, resulting in water levels of 4.1 ppm or less [59]. Additional activated molecular sieves reside in the solvent reservoir of the SAC to continue active drying during subsequent solvent vapor anneals. Following solvent vapor exposure and complete solvent evaporation from the film, the samples were immediately transferred to a 0.05 M NaOH solution (H_2_O:CH_3_OH = 6:4 by volume) for PLA minority domain degradation and left to soak for 45 min, where the degradation rate is sensitive to molarity [60]. After removal, films were washed with deionized water/methanol for 5 min. By removing the minority component immediately, this serves to ensure the morphology of the film is immobilized for subsequent imaging. Furthermore, to remove any additional surface contamination, a 150 W, 50 KHz O_2_ reactive ion etch (PE-50, PlasmaEtch, Inc., Carson, NV, USA) is employed for 10 s at 100 mTorr. This process removes ~2–3 nm of organic material. Samples at this stage were immediately imaged with atomic force microscopy (AFM), without any further modification.

### 2.4. Measuring Film Thickness

Film thickness was determined with a Filmetrics F20-UV (San Diego, CA, USA) general-purpose film thickness measurement system with both halogen and deuterium sources. Spectral reflectance data was taken at differing time intervals (as discussed below), between 10 ms–3 s with a 10–250 ms integration time. Experimental data was modeled over a spectral range of 270–900 nm with a three-layer model (Si + PS-*b***-**PLA + air). We developed anticipated refractive index profiles based on known values (e.g., PS, n = 1.59; PLA, n = 1.482; THF, n = 1.407). Therefore, we expected an index of refraction of 1.55 for the neat film, dropping to 1.45 with increasing solvent (THF) concentration up to φ = 0.55 [34]. Samples not following this trend in refractive index with increasing solvent were aborted and disposed.

### 2.5. Atomic Force Microscopy (AFM)

Tapping mode AFM was performed on an Agilent 5420 microscope (Santa Clara, CA, USA) under ambient conditions using engagement setpoints between 0.9–0.95 of the free amplitude oscillation. The tapping mode cantilevers (BudgetSensors, Sofia, Bulgaria and Bruker, Billerica, MA, USA) had a resonant frequency of 300 kHz and a force constant of 40 N/m. For imaging the film/substrate interface, the PLA-removed thin films were placed upside down on double-sided transparent tape (ScotchBrand, St. Paul, MN, USA) and placed in liquid N_2_ for 30 s. Following liquid N_2_ exposure, the Si wafer was peeled away from the film providing access to the underside. These films were again exposed to a 10–20 s O_2_ reactive ion etch (150 W 50 KHz in 100 mTorr) on the underside to remove any HMDS, a thin PS wetting layer or adhesive residue [34,61].

## 3. Results

### 3.1. Design of a Purpose-Built Solvent Vapor Annealing Chamber

The reproducibility of a final morphology and its propagation into solvent vapor-annealed BP films remains somewhat elusive, despite numerous advances in the field. To directly address this issue, we have designed, constructed and tested a purpose-built solvent vapor annealing chamber (SAC) that provides unparalleled control over introducing and maintaining precise solvent concentrations within the film during the annealing process as well as during the evaporation of solvent from the film. Concurrently, we maintain control over several additional necessary parameters (Table 1), including an exceptionally low dew point in the sample cell with active dry N_2_ purging, solvent vapor flow rate, film thickness as a proxy for solvent concentration in the film [34], chamber pressure, solvent selectivity, and substrate surface preparation. The present investigation uses ~60 nm thick films of a prototypical BP, PS-*b*-PLA, that adopts a cylindrical morphology in the bulk. We use at room temperature a relatively neutral solvent, THF, for the anneal [29,34,62]; a PS-selective substrate, HMDS-functionalized Si; and maintain a low dew point (−100 °C) for the N_2_ carrier gas, due to the hygroscopic nature of THF and PLA. The following SAC description is a significant evolution over initial work in developing these protocols, including the addition of pneumatically-actuated, computer controlled solvent flow rates and valve actuation [34,38]. In our method, a copper-gasket sealed, all-metal chamber controls the SVA climate (i.e., humidity, chamber pressure, and evaporation times). The all-metal construction, except for chemically inert modified polytetrafluoroethylene sealed ball valves and a perfluoroelastomer sealed low-flow metering valve, ensures negligible interaction with the solvent during the annealing process. In particular, THF can be particularly aggressive on traditional organic gaskets and lubricants. Following hundreds of hours of exposure to THF, no degradation to any valves in the chamber is evident.

**Figure 2:**
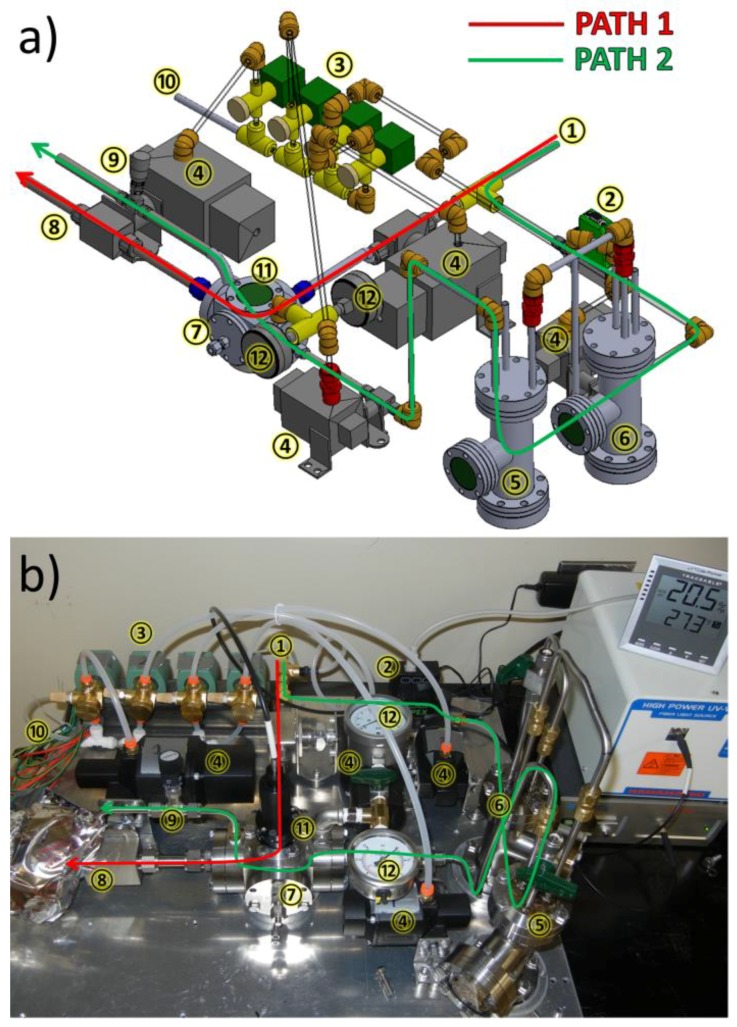
(**a**) Computer-aided design (CAD) and (**b**) real image of a computer-controlled, pneumatically actuated solvent vapor annealing chamber. More images can be found in Appendix A. Numbers and colored arrows are described in the text.

The chamber, shown in Figure 2 (additional images are available in Appendix A), is connected to an actively dried N_2_ line (dew point guaranteed to −100 °C) ①, which splits into two 1/4” stainless steel tubing paths. Path 1 (in red) purges the sample space before annealing and during sample loading; this ensures a low dew point during the SVA. Additionally, BP films are hot-loaded from a 75 °C oven into the purged, over-pressurized chamber via a Conflat-flanged door and immediately sealed to avoid water contamination and allowed to cool to room temperature. Path 1 is also vital to the controlled evacuation of solvent vapor from the SAC during the evaporation phase. N_2_ flow is passed through an acrylic, block-style flowmeter (3–30 SCFH or 30–300 SCFH, dependent on flow rate chosen) to control and measure flow rates during solvent evaporation.

**Figure A5:**
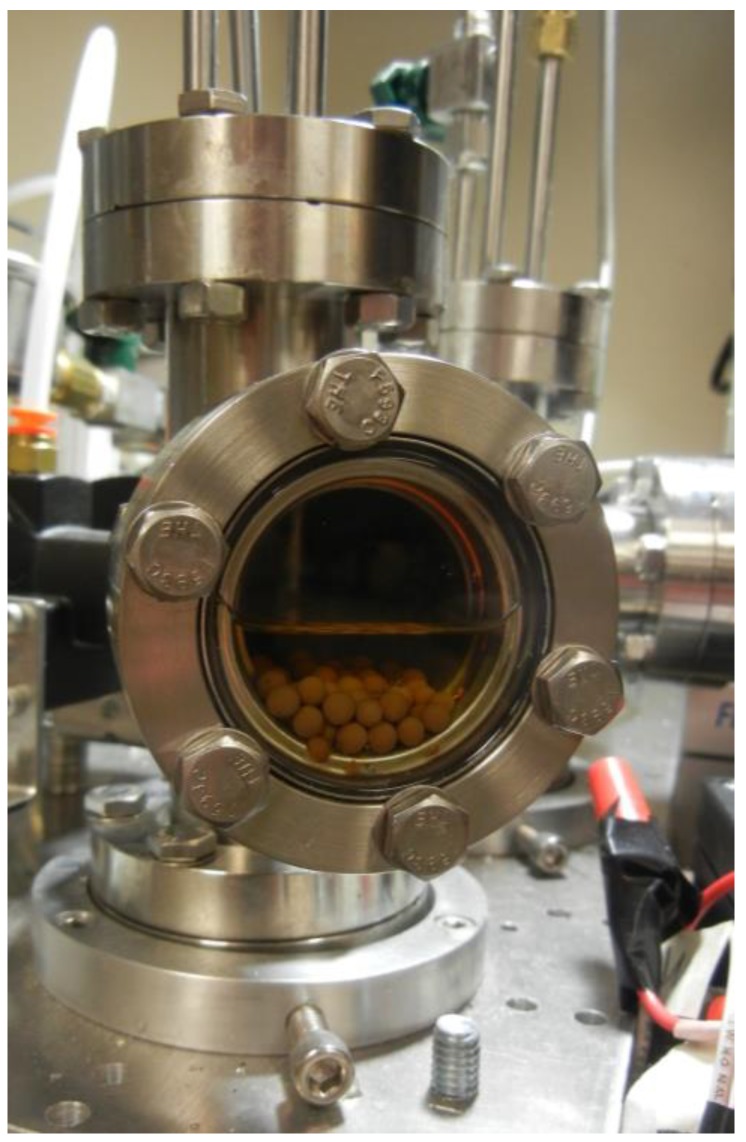
Solvent reservoir with optical view port, liquid solvent, and molecular sieves.

**Figure A6:**
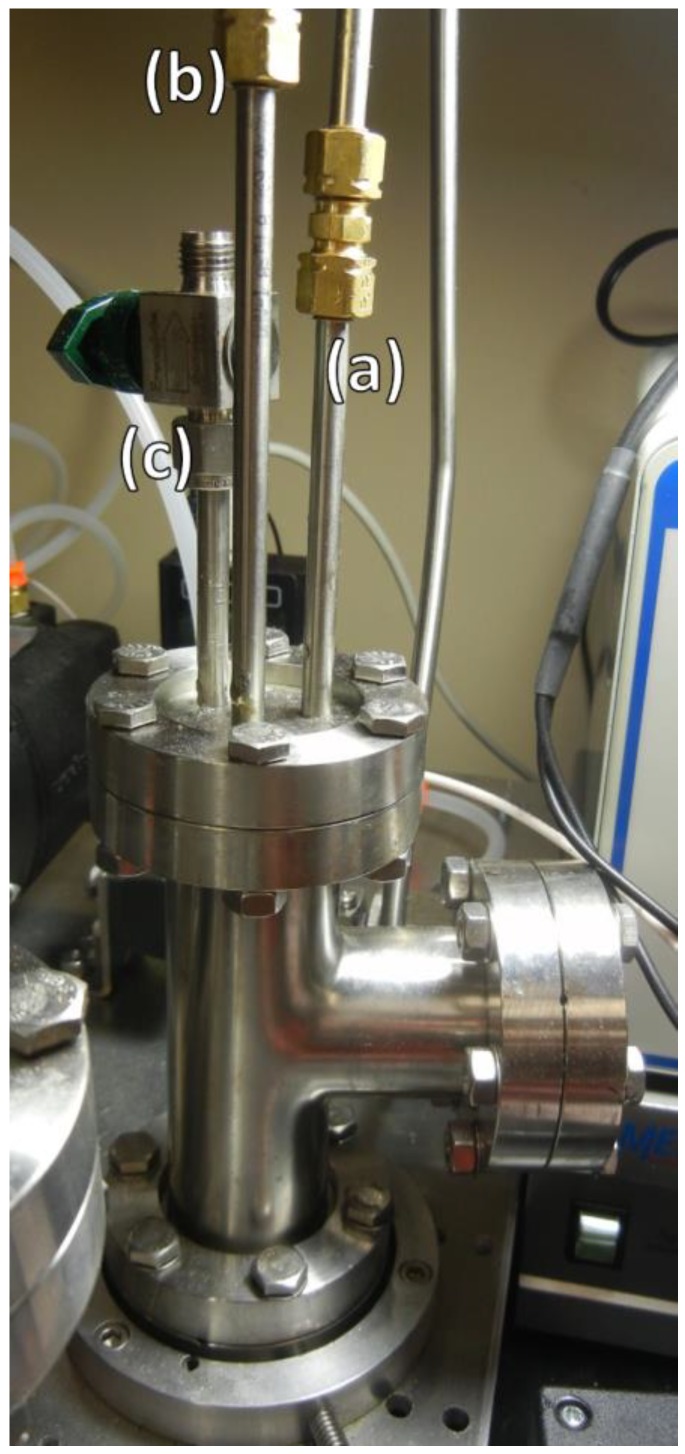
Depiction of the solvent reservoir with three ¼” tubing ports. One tube (**a**) is an N_2_ inlet that extends to the bottom of the reservoir. One tube (**b**) is a solvent vapor outlet that only extends into the reservoir ½” to capture the vapor. One tube (**c**) is capped with a normally-closed plug valve. This is used to add liquid solvent without opening the Conflat flanges.

Path 2 (in green) flow is governed by a mass flow controller ② connected to a LabVIEW-enabled computer (National Instruments, Austin, TX, USA). Flow rates range from 0–500 SCCM, dependent on intended anneal conditions. The flow of the metered, actively dried N_2_ gas is computer controlled via solenoid actuated pneumatic valves ③ that control 80 psi (551 kPa) air flow to each of four pneumatic process valves ④. Following the flow controller, the N_2_ gas is passed through a primary safety valve ④ (this protects the mass flow controller from liquid solvent exposure). The controlled gas flow continues on its way to a sealed, molecular-sieve-dried solvent reservoir ⑤, a stainless steel Conflat tee with view window (Figure A5). This reservoir contains an additional inlet tube with a normally closed plug valve that is used for liquid solvent loading without breaking any Conflat seals Figure A6). The solvent reservoir is backed by a safety reservoir ⑥, a stainless steel Conflat tee with viewing window. The safety reservoir will collect fluid solvent if backpressure is present, avoiding exposure to the mass flow controller. The safety reservoir contains an additional tube with a normally closed plug valve to remove any liquid solvent without opening any Conflat seals. Ultimately, the dry N_2_ is bubbled through the solvent reservoir ⑤, which subsequently carries solvent in the vapor phase into the sample space ⑦, a 6-way Conflat cube (70 mm × 70 mm × 70 mm), via a computer-controlled, pneumatically-actuated chamber valve ④. All flow into the sample space exits through 3/4” stainless steel tubing via a pneumatically-actuated ball valve ⑧ or a low-flow metering valve ⑨ to a fume hood. The computer controlled, pneumatically-actuated valves ④, with compressed air inlet ⑩ and 120 V solenoid valves ③, allow us to quickly initiate and terminate the SVA with a specified level of timing. Of critical importance, initial testing indicates a controlled SVA evaporation time down to 15 ms or any time longer with 10–20 ms temporal resolution. To our knowledge, this is the fastest recorded SVA evaporation time for a BP thin film.

**Figure A7:**
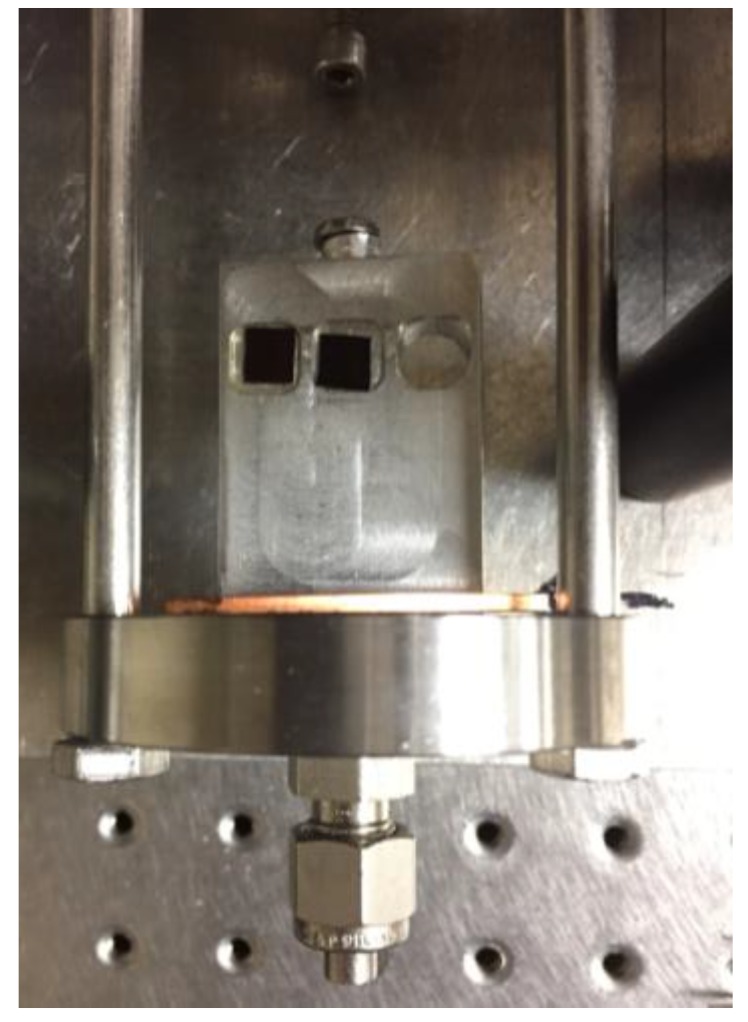
Custom designed sample holder. The block copolymer film sits in the center well, a silicon optical standard sits in the left well. A hole for background ultraviolet-visible (UV-V) light is spectroscopy subtraction is on the right. The sample holder rests on a two-pronged frame to slide the samples in and out of the main chamber and is sealed with a Conflat flange with copper gasket.

The BP sample resides in the sample space ⑦ on a custom-built mount (Figure A7). The sample mount features two recessed ports, which speeds sample loading and helps avoid direct flow from Paths 1 and 2 that can shift sample position. One recessed port holds the BP film, while the other holds a blank Si wafer for use as an optical standard. Recess ports are sized to hold up to an 8 mm × 8 mm BP film and a blank Si wafer. The chamber offers direct optical access to the sample space through a fused silica viewport ⑪, enabling continuous monitoring of the sample chamber with high precision pressure gauges ⑫ to directly measure chamber pressure and in situ spectral reflectance-based measurements of film thickness with 0.1 nm thickness and 10–20 ms temporal resolution. These thickness measurements are directly related to solvent volume concentration (φ) within the film by:

(1) $$\phi =\frac{V_{solvent+film}-V_{film}}{V_{solvent+film}}=\frac{t_{solvent+film}-t_{film}}{t_{solvent+film}}=1-\frac{t_{film}}{t_{solvent+film}}$$

where φ is solvent concentration, *V* denotes volume and *t* is film thickness. As supported by direct observation, the areas for the two film states (i.e., swollen versus dry) are taken to be nominally identical and thus only a thickness measurement is required to obtain a real-time in situ probe of φ during SVA.

### 3.2. Theory of Operation

**Figure 3:**
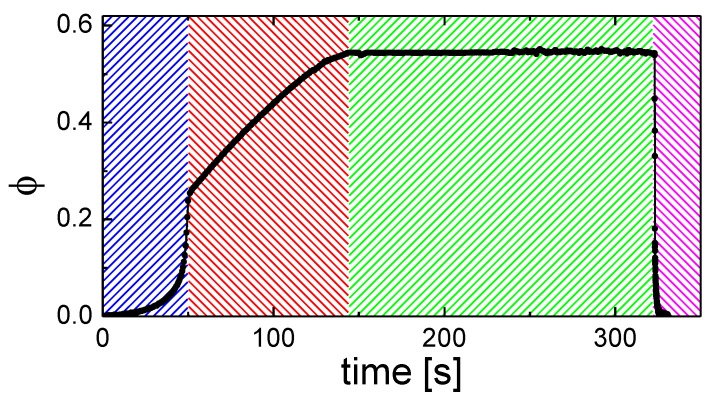
Solvent concentration in a BP film (ϕ) versus anneal time for a typical solvent vapor anneal of a 60 nm PS-*b*-PLA thin film. The blue indicates the initial solvent uptake regime. The red indicates the metered solvent uptake regime. The green indicates a fixed solvent concentration regime. The magenta indicates the solvent evaporation regime.

**Figure A8:**
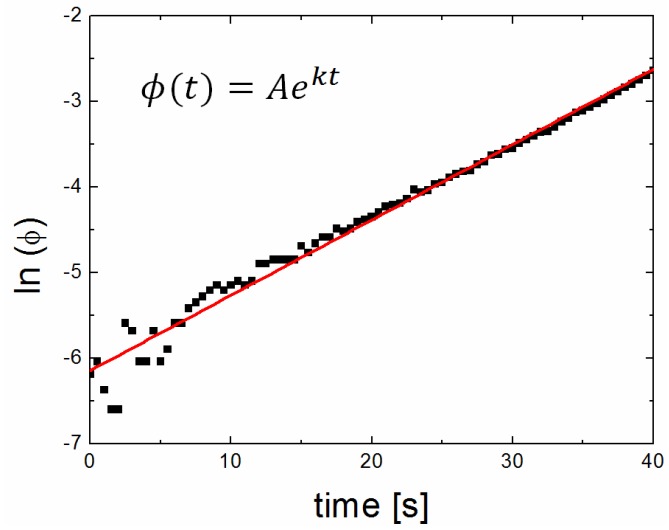
Best linear fit to the natural log of solvent concentration for the first 40 s (regime 1) of Figure 3, showing a single exponential rate constant of 0.088 ± 0.0013 s^−1^. The R^2^ value of the fit is 0.982.

**Figure A10:**
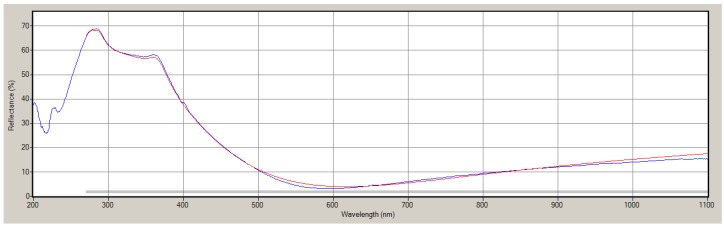
Spectral reflectance of a THF swollen polystyrene-*block-*polylactide film on Silicon. The film contains 55% THF solvent. The blue line is reflectance data; red line is the spectral reflectance model using an index of refraction of 1.45 for the copolymer layer.

Through the active control of solvent vapor inlet and outlet flows, the solvent concentration within the BP film is controlled and monitored as a function of time. As shown in Figure 3, using an in situ measurement of φ, we divide the SVA process of a 60 nm PS-*b*-PLA film into four distinct time regimes. During the first three time regimes, thickness data was taken every 3 s with a 249 ms integration time. The initial regime (blue) includes the opening of the solvent reservoir to the sample chamber and an initial, constant N_2_ inlet flow of 30–100 SCCM (dependent on desired chamber pressure, 50 SCCM in the present study), resulting in an exponential increase in ϕ. The thickness data is well modeled assuming a copolymer refractive index of 1.55, consistent with the neat PS-*b*-PLA film (Appendix B). This exponential region typically lasts ~60 s where the time dependence is well modeled with a single rate constant (0.03–0.1 s^−1^) with 0.088 s^−1^ for Figure 3 (see Figure A8 for the exponential fit), which is dependent on exact inlet and outlet flow rates. Outlet flow rates are governed by a low flow micrometer relief valve, which is set to a flow coefficient, *C_v_*, of 0.0002–0.002 (1–6 turns), with 0.0004 being used in Figure 3. This, along with inlet flow, dictates the chamber pressure, which was maintained below 3.5 kPa to obtain Figure 3. Following the displacement of residual N_2_ in the chamber with solvent vapor, the solvent uptake enters a second regime (red). This regime involves the metered uptake of solvent into the film, indicated by a controlled, linear increase in thickness over a period of 2 min (highly tunable, based on relative flow rates), until the film reaches the targeted φ. The inlet and outlet flow rates remained the same for the second regime as given for the first regime in Figure 3, demonstrating this change in solvent uptake. The thickness data is well modeled assuming a copolymer refractive index that gradually approaches 1.45, consistent with a volumetric combination of neat PS-*b*-PLA and THF (Appendix B). During this phase, two possible methods drive solvent into the film and increase the thickness. If the inlet flow is higher than outlet flow, the increasing pressure in the chamber will increasingly force solvent into the film (a relatively fast mechanism). If the inlet and outlet flows are comparable, the increase in relative solvent concentration within the chamber leads to an increased uptake of solvent into the film (a relatively slow mechanism). Therefore, it is possible to swell a film with a chamber pressure that is nominally atmospheric. Dependent on desired chamber pressure and ultimate thickness, the inlet and outlet flow rates are tuned. Precise control is best achieved by adjusting the mass flow controller-regulated inlet. We have not seen any impact with the rate of swelling in this second regime on the final morphology of the film, other than a potential dependence on the pressure in the chamber (faster swelling is typically accomplished with an increase in chamber pressure). The thickness data in this regime is well modeled assuming a copolymer refractive index of 1.45 (Figure A10), consistent with a solvated PS-*b*-PLA film containing φ = 0.55.

**Figure 4:**
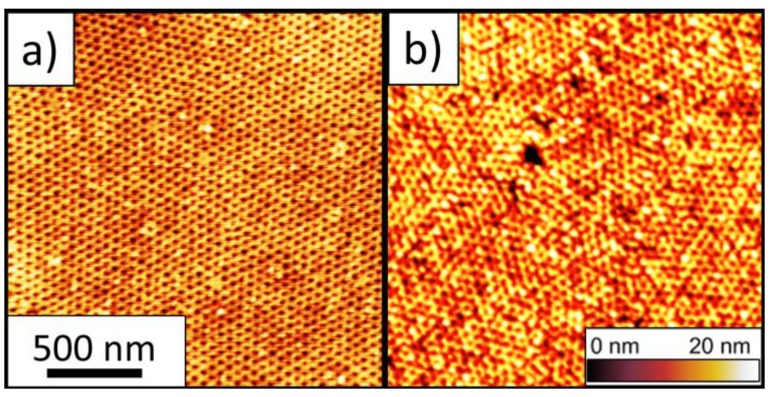
Atomic force topography micrographs of the free surface (**a**) and substrate surface (**b**) of a 60 nm PS-*b*-PLA film that was exposed to a THF concentration φ = 0.55 and having a solvent evaporation occurring in 15 ms. Images were taken following hydrolytic removal of the PLA minority component and a brief (10 s) O_2_ reactive ion etch. The false color height scale is 20 nm.

As the target φ is approached, decreasing the inlet flow or increasing the outlet flow causes φ to level off. The third regime (green) is characterized by an extremely constant (standard deviation is regularly less than Δφ = 0.002) solvent concentration. This constant concentration can be maintained for nearly any specified anneal time (3 min is shown in Figure 3, but we have maintained similar consistency for more than an hour). It is maintained through slight manual variations in inlet/outlet flow rates, with the future potential of computer feedback control. Figure 3 was achieved with a fixed outlet flow, consistent with regimes 1 and 2 and by controlling the inlet flow between 10–30 SCCM to achieve a constant thickness (solvent concentration). In the final seconds of this period, the integration time and thickness acquisition interval are switched to 10 ms and 0 s, respectively. This is the fastest we can acquire spectral reflectance data and still get a high-fidelity model to extract thickness. This rapid data acquisition allows for close examination of the solvent evaporation period. In the fourth regime (magenta), evaporation of the solvent from the film occurs. Computer-controlled, pneumatically-actuated valves open Path 1 and close Path 2. Through the release of pressure in the chamber, in tandem with N_2_ flow, the solvent vapor is released from the film and evacuated from the chamber. Details on timing control, which are dependent on Path 1 flow rates and chamber pressure, are given below. The initial evaporation of solvent from the film is not complete, where φ ≈ 0.2 typically remains in the film over the first 100 ms. This remaining solvent typically takes an additional 1.5–10 s to be fully removed from the film, dependent on the Path 1 inlet flow rate. At φ < 0.25–0.3, the film is likely vitrified with exceptionally slow kinetics, consistent with recent GISAXS results [32]. Therefore, the final morphology is well locked-in during the initial, potentially fast, evaporation phase. In fact, atomic force micrographs of the free surface and the substrate surface (after removal from the Si substrate) of a PS-*b*-PLA film verify that a vertically-aligned cylindrical morphology can persist through an entire 60 nm film to the substrate surface (Figure 4). The PS-*b*-PLA film in Figure 4 was swollen with THF to φ = 0.55 and the solvent was subsequently evaporated out of the film (down to φ = 0.2, sufficient to vitrify the film) in 15 ms. No condensation of solvent was observed during any changes in pressure for the present investigation.

### 3.3. Solvent Evaporation

**Figure 5:**
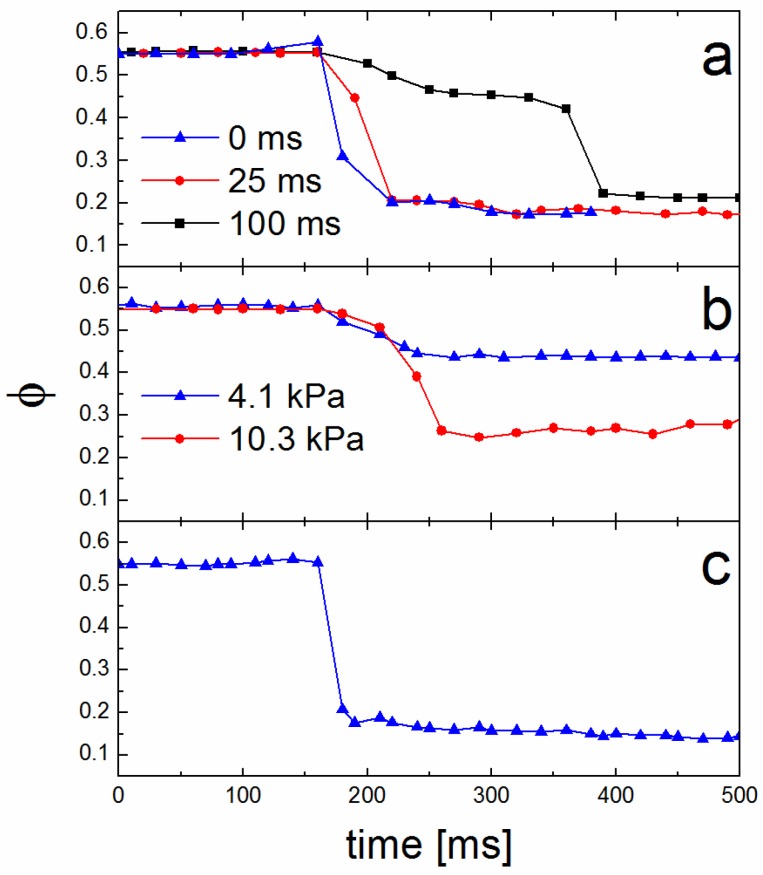
Solvent concentration (ϕ) versus anneal time during solvent evaporation; (**a**) solvent evaporation trajectories for three time delays between opening the chamber outlet to atmosphere and introducing an inlet flow of N_2_; (**b**) solvent evaporation trajectories for an annealing chamber with a relatively high or low vapor pressure; (**c**) Solvent evaporation trajectory for a fast evaporation with high inlet flow and zero vapor pressure in the chamber.

While a primary outcome of this purpose-built chamber is the ability to keep an arbitrary solvent concentration constant within a BP film for a specified time, another beneficial consequence is the ability to observe and control the solvent evaporation phase (fourth regime—solvent evaporation magenta regime—in Figure 3) with 10–20 ms temporal resolution. This high acquisition rate has revealed subtle differences in precise evaporation trajectories, dependent on exact chamber pressure and inlet/outlet flow rates. First, of importance to our specific chamber, is the timing between the inlet and outlet valves of Path 1. It is necessary to introduce a slight time delay between their actuation. This is detailed in Figure 5a. If the valves are open concurrently (i.e., 0 ms time delay), the finite impedance of the outlet tube causes a brief pressure spike in the chamber that drives residual solvent vapor from the chamber into the film. This increases the concentration within the film, possibly to disorder, ahead of the evaporation. This leads to inconsistent morphologies and is undesirable. If the outlet valve is opened far ahead of the inlet valve, a two-stage evaporation tends to take place. The opening of the outlet valve releases pressure from the chamber and leads to a drop in concentration within the film. Presumably, some solvent was retained in the film simply due to the finite pressure in the chamber during the anneal. Then, the opening of the inlet flow removes the remaining solvent in the chamber and the solvent is fully evaporated from the film. For example, a delay of 100 ms has two distinct evaporation trajectories, as seen in Figure 5a. For our specific system, a computer-controlled time delay of 25 ms leads to optimally fast evaporation without the associated pressure spike (Figure 5a).

Second, considering the role of chamber pressure during the evaporation phase; the release of pressure within the chamber as the outlet valve of Path 1 is opened leads to a release of solvent from the film. This is true even in the complete absence of any inlet N_2_ flow, as shown in Figure 5b. As expected, the higher the pressure in the chamber, the more dramatic the decrease in solvent concentration in the film when the outlet valve is opened. In the case where pressure in the chamber approaches 10.3 kPa, the pressure drop to 0 kPa is sufficient to remove solvent down to φ = 0.25. This concentration is sufficiently low to lock-in the morphology where only kinetically slow vitrification persists, discussed above. In the case where chamber pressure is somewhat lower, 4.1 kPa, the release of pressure to 0 kPa is insufficient to remove a sufficient amount of solvent to vitrify the film without additional inlet N_2_ flow. Finally, considering inlet and outlet flows (Figure 5c), a higher Path 1 inlet flow rate leads to optimally fast solvent vapor removal from the chamber. This potentially leads to rapid solvent evaporation from the film (10–20 ms). This rapid evaporation causes a strong ordering front and tends to drive cylinder propagation perpendicular to the film (Figure 4). After reviewing 700 trials following these SVA protocols, including rapid evaporation (~15 ms) and low chamber pressure, 640 (91.4%) films showed full perpendicular alignment, 14 (2.0%) showed full in-plane alignment, and 46 (6.6%) exhibited a mixed, mostly perpendicular alignment with some in-plane cylinders evident. The latter two cases (in-plane cylinders or mixed) were regularly attributed to elevated ambient humidity in the lab. Clearly, the control over both chamber pressure and Path 1 flows provide extensive control over the solvent removal rate and its specific trajectory. The resultant morphologies and their propagation into the bulk of a film for these different trajectories are the subject of a forthcoming manuscript.

## 4. Conclusions

It is increasingly clear that annealing BP thin films is critical to achieving self-assembled nanostructures with a given morphology and lateral order. Solvent-based techniques have proven to be highly effective due to the dramatically increased polymer chain mobility while mitigating thermal degradation. In particular, chain mobility near the order disorder transition is optimally enhanced. In addition, the evaporation of solvent from a film is critical in determining the propagation of a given morphology into the bulk of the film. At present, there are three primary methods for incorporating solvent into a BP film; however, each is limited in its ability to directly control solvent concentration within the film and the solvent evaporation rate. These limitations have stifled investigation into the time-dependence of these effects. Furthermore, solvent-enhanced crystallization has evolved from BPs to other organic systems, with conjugated polymer/organic photovoltaics being one illustrative example [63], further indicating its potential efficacy. Therefore, we have presented a purpose-built solvent vapor annealing chamber that was designed and constructed to elucidate the role of key parameters involved in directed self-assembly in BP thin films with goals of enhanced reliability, repeatability and the eventual scaling up of the SVA process. Currently, the use of in situ scattering techniques, such as GISAXS or GISANS, are critical for understanding the ordering mechanisms of block polymer films. However, they may prove impractical for industrial applications. Therefore, transferring protocols developed with those techniques to purpose-built chambers, such as the one presented here, could be essential for proliferation. In particular, there is interest in observing and controlling the mechanisms for increased correlation lengths of self-assembled features and the growth propagation of those features into the bulk of the film in the final film state (i.e., following solvent evaporation). This level of control opens up possibilities for a variety of morphology controls. Moreover, in contrast to many current efforts, the technique does not involve extensive in situ monitoring with advanced scattering techniques, but could serve to enhance those techniques with higher temporal resolution and control. Rather, the present in situ monitoring relies on inexpensive, readily available optical techniques in conjunction with pneumatically-actuated, computer-controlled flow controllers and valves. Such low-cost and simple methods could prove useful for any future scaling-up of this process.

## Figures and Tables

**Table 1:** A compilation of variables considered in the design of solvent vapor annealing protocols, along with their importance and the steps taken in the design to address them.

Variable	Importance	Protocols in Design
Humidity	Water is a polar solvent, which will modify the solubility. For example, water is PLA selective	Samples are stored in 75 °C oven Samples are hot loaded into chamber Vacuum-grade, copper gaskets and Swagelok seals throughout chamber Molecular sieve-dried solvent (tetrahydrofuran (THF)) Actively purged (dry N_2_) sample cell Drierite Gas Purifier (−100 °C dewpoint)
Solvent Vapor Flow Rate	Flow rate is proportional to solvent uptake in film	Computer-controlled mass flow controller Low-flow metering outlet valve
Solvent Concentration	Solvent concentration in film during solvent vapor annealing (SVA) modifies mobility	In situ optical detection of solvent concentration, assuming proportional to film thickness, with 10–20 ms resolution
Solvent Evaporation Rate	Evaporation rate in linked to morphology alignment	Computer-controlled pneumatic valves Variable flow N_2_ purge line
Initial Thickness	Role of commensurate thickness on morphology	Films are all spun cast at a constant spin speed from the same solution concentration
Vapor Pressure	Vapor pressure during SVA modifies solvent uptake and evaporation	High and low pressure gauges Low-flow metering outlet valve can finely adjust vapor pressure
Solvent Selectivity	Solvent selectivity modifies surface energy and polymer-polymer interactions	THF is a relatively neutral solvent for PS and PLA. It has slight PS selectivity
Surface Selectivity	Substrate preparation modifies surface energy	HMDS-functionalized Si substrate surface promotes PS (majority block) adhesion

**Figure A1:**
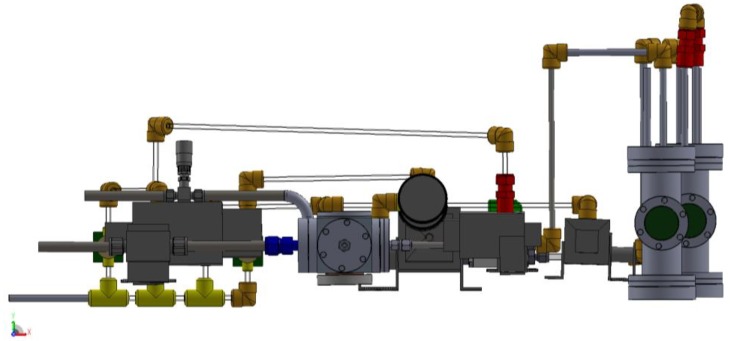
Front view of the solvent vapor annealing (SVA) chamber.

**Figure A2:**
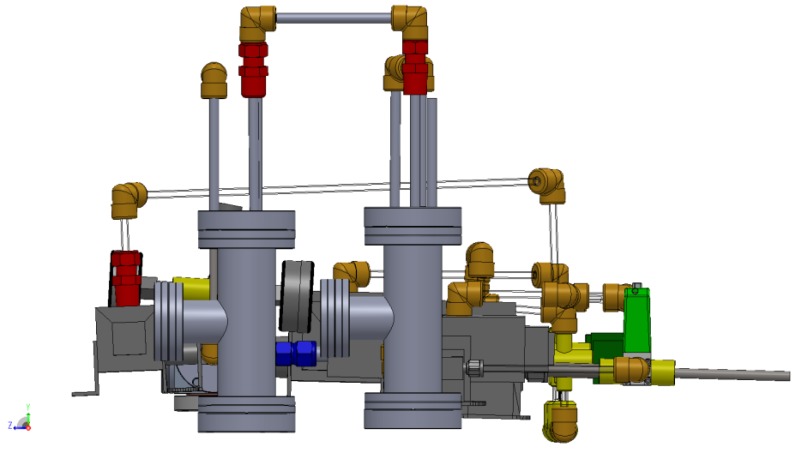
Right view of the SVA chamber.

**Figure A3:**
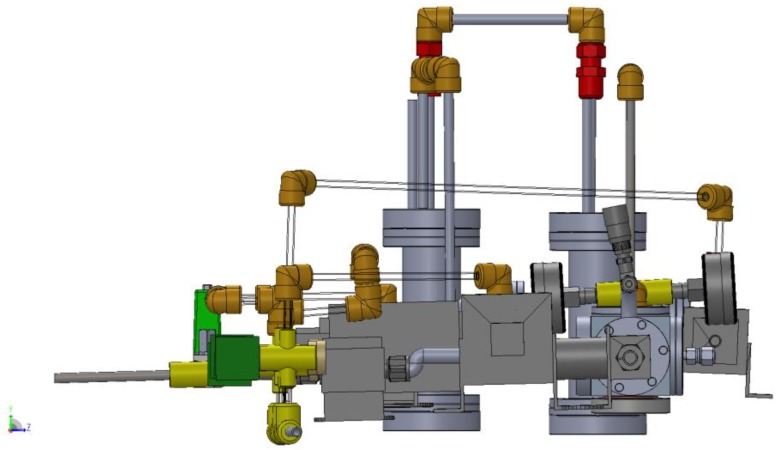
Left view of the SVA chamber.

**Figure A4:**
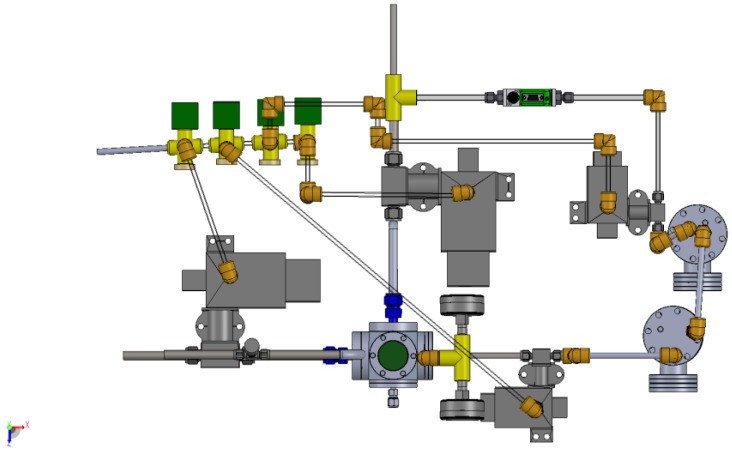
Top view of the SVA chamber.

**Figure A9:**
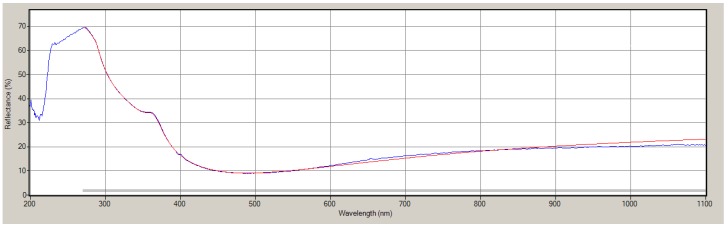
Spectral reflectance of a neat 60 nm polystyrene-*block*-polylactide film on silicon. The blue line is reflectance data; red line is the spectral reflectance model using an index of refraction of 1.55 for the copolymer layer.
